# Investigating the role of attentional effort in the efficacy of goal-setting in reducing attention lapses

**DOI:** 10.3758/s13414-025-03200-9

**Published:** 2025-12-04

**Authors:** Deanna L. Strayer, Nash Unsworth

**Affiliations:** Department of Psychology, University of Oregon, Eugene, OR 97403 USA

**Keywords:** Attention, Cognitive and attentional control

## Abstract

Attention lapses occur when focus shifts away from the task at hand towards internal or external distractions and can lead to failures in completing intended actions. Goal-setting theory proposes that setting specific, difficult goals leads to better task performance over vague goals. The present study examined whether goal setting increased attentional effort and reduced attention lapses during a four-choice reaction time task. The control condition received the vague goal: “respond as quickly as possible while keeping your accuracy above 95%.” The goal condition received specific goals that became progressively harder over time (450 ms, 400 ms, and 350 ms) with the same accuracy goal. Pupillary responses were recorded throughout and subjects answered randomly presented thought probes to determine whether they were experiencing task-unrelated thoughts (TUTs). The goal condition displayed larger preparatory and phasic pupil responses, suggesting more attentional effort was exerted during the task. In addition, the goal condition displayed fewer attention lapses both behaviorally and with TUTs. Further, several typical time-on-task effects were mitigated or eliminated (shown in behavioral, subjective, and physiological measures). The results reinforce previous findings that goal-setting techniques can reduce attention lapses and indicate attentional effort is a mechanism behind the efficacy of goal setting.

## Introduction

Sustained attention entails maintaining focus on a task for time periods that can span from seconds to hours. This ability is a crucial element of our attentional system that makes us able to go about day-to-day life. Various cognitive and conative factors such as motivation, arousal, and alertness greatly impact sustained attention (Jennings & van der Molen, 2005; Sadaghiani & D’Esposito, 2015; Steinborn et al., 2017; Unsworth & Miller, 2021). Additionally, strategies such as rest breaks can promote the restoration of attentional capacity (Schumann et al., 2022). Some tasks that require sustained attention can be monotonous and unchallenging, making them harder to maintain focus on compared to cognitively engaging tasks (also referred to as vigilant attention; Langner & Eickhoff, 2013; Lim & Dinges, 2008; Robertson et al., 1997; Robertson & O’Connell, 2010; Warm, 1984). Despite our general ability to perform well in sustained attention scenarios, occasional attention lapses are inevitable. Attention lapses indicate momentary shifts of focus away from the task at hand and can lead to failures in completing intended actions (Casner & Schooler, 2014; Lindquist & McLean, 2011; Unsworth et al., 2021; Unsworth & McMillan, 2017). These lapses can manifest as daydreaming, environmental distractions, or even moments of mind-blanking. Understanding the nature of attention lapses and investigating methods to reduce their frequency and severity is essential due to their potential consequences, ranging from minor oversights to severe accidents.

Goal-setting has been shown to be a promising approach in the search for ways to mitigate the occurrence of attention lapses. According to goal-setting theory, specific and difficult goals can enhance performance (Locke & Latham, 1990, 2002, 2019). Indeed, prior research in the realm of sustained attention tasks has indicated that such goals can lead to improved task performance and fewer attention lapses, particularly when the specific goals became progressively harder over the course of the task (Robison et al., 2021; Strayer et al., 2024). However, the mechanisms underlying these improvements are not fully understood.

The primary goal of the current study was to determine whether attentional effort, as indexed by pupillometry, is related to the previously observed reduction in attention lapses with progressively harder over time (HOT) goals (Strayer et al., 2024). By examining phasic and preparatory pupil responses, we aimed to capture physiological correlates of changes in attentional effort and arousal. This study integrated behavioral performance (response times; RTs), subjective reports (task-unrelated thoughts; TUTs), and physiological measures to deepen our understanding of the mechanisms through which goal setting impacts sustained attention and attention lapses.

## Attention lapses

The primary behavioral method for studying attention lapses involves examining RTs and RT variability as indicators of attentional fluctuations. Longer RTs are often interpreted as signs of lapses in attention. Bills (1931) first introduced the concept of “blocks” after finding that unusually long RTs (greater than twice the mean RT within subject) would crop up during homogenous mental tasks and that they became more frequent over time. His conceptualization of this phenomenon was that the subjects’ minds “blocked” them from being fully present during some trials as a way to take mini-breaks and counteract fatigue (Bills, 1931, 1935). Later research by Bertelson and Joffe (1963) supported this notion, observing that these “blocks” were preceded by increasing RTs and errors, but followed by improved performance, indicating a restorative effect. Broadbent (1958) took a different stance and posited that “blocks” are shifts of focus towards task-irrelevant stimuli.

Contemporary studies conceptualize these long RTs and RT variability as attention lapses (Dinges & Powell, 1985; Esterman et al., 2014; Steinborn et al., 2016; Unsworth & Robison 2016, 2020). Esterman et al. (2014) used the gradual-onset continuous-performance task to show that long RTs correlated with decreased vigilance and increased mind wandering. Further, Van Breukelen et al. (1995) utilized the entire RT distribution to examine attention lapses on the assumption that some trials will represent instances where the subject was focused (i.e., fast RTs) and some will indicate subjects experiencing a block/lapse (i.e., longer RTs).

However, several studies have noted that these methods are an imperfect measure of attention lapses (Dinges & Powell, 1985; Steinborn et al., 2016; Unsworth & Robison, 2016; Van Breukelen et al., 1995; Weissman et al., 2006; Williams et al., 1959). Interpreting RT data can be complex because, while lapses likely contribute to RT variability and long RTs, some portion of the effect can be attributed to factors such as eye movements/blinks, inter-trial interval timing, speed-accuracy trade-offs, and individual differences in response strategies (Johns et al., 2009; Steinborn & Langner, 2012; Unsworth et al., 2021). Additionally, if considered to be lapses, impulsive reactions to stimuli resulting in overly fast RTs can affect the distribution (Kane & McVay, 2012; Unsworth, 2015).

In addition to behavioral measures, subjective methods such as thought probes have been used to assess participants’ thoughts during a task and whether those thoughts are task-related or unrelated (see Smallwood & Schooler, 2015). Thought probes involve periodically interrupting the task to ask participants about their current thoughts, distinguishing between task-related thoughts (TRTs) and task-unrelated thoughts (TUTs) (Giambra, 1995; Smallwood & Schooler, 2006). Research has consistently found that TUTs negatively correlate with task performance, with higher rates of TUTs associated with increased attention lapses (McVay & Kane, 2010; Mooneyham & Schooler, 2013; Smallwood & Schooler, 2006; Unsworth & McMillan, 2014).

Further categorization of TUTs into mind-wandering, external distractions, and mind-blanking provides additional insights into the nature of attention lapses (Stawarczyk et al., 2011; Unsworth & Robison, 2016; for review, see Smallwood & Schooler, 2015). For example, mind-wandering involves internally focused thoughts unrelated to the task, while external distractions refer to shifts in attention toward irrelevant external stimuli. Mind-blanking, characterized by a lack of conscious thought, represents a more extreme form of disengagement from the task.

Pertinently, recent studies by Unsworth et al. (2021, 2022; see also Kucyi et al., 2016, and Welhaf & Kane, 2024) suggest that while behavioral (RT) lapse measures and subjective (TUT) measures share considerable overlap, they also have some unique characteristics. They seem to capture different aspects of attention lapses. Using both measures can help provide a more comprehensive picture of lapses, but they are not interchangeable measures.

## Goal setting

Industrial-organizational psychology research suggests that setting specific, difficult (but achievable) goals effectively improves task performance (Locke & Latham, 1990, 2002, 2019; Robison et al., 2021). Two key components of goal setting are content and intensity (Locke & Latham, 1990). The content component includes goal-specificity, which spans from vague (“do your best”) to specific (“keep your responses under .400 s while keeping your accuracy above 95%”). Content also includes goal-difficulty, which is dependent on the abilities/motivation/commitment of the individual engaging in the task and which spans from overly easy to impossible to achieve. Setting goals that are specific and difficult, while remaining achievable, is found to improve performance compared to vague goals (Locke & Latham, 1990). Locke and Latham (1990, 2002) have laid out four ways in which goals affect performance: (1) they concentrate attention and effort towards the task and away from TUTs; (2) they increase the intensity of effort and attention allocated to the task; (3) they perpetuate persistence and effort; and (4) they stimulate self-development of performance increasing strategies. Put simply, specific goals serve to increase intensity and persistence of attentional effort (Locke & Latham, 1990).

Robison et al. (2021) explored these theories in the context of sustained attention, hypothesizing that setting a specific goal during the psychomotor vigilance task (a simple RT task; Dinges & Powell, 1985) would improve sustained attention performance and reduce attention lapses compared to a vague goal. Two of their experiments found evidence that specific goals speeded the slow tail of the RT distribution, but this effect was not replicated in another which included a range of goal levels. Robison et al. (2021) also examined the vigilance decrement, a time-on-task effect where performance tends to worsen over time during sustained attention tasks (Parasuraman, 1986; Parasuraman & Davies, 1977; Mackworth, 1950; Robison et al., 2021; See et al., 1995). Again, two experiments found the vigilance decrement was attenuated, providing evidence that goal setting may improve overall sustained attention. Similarly, Strayer et al. (2024), used a four-choice RT task and found across three experiments that setting specific goals reduced lapses in the slow tail of the RT distribution, particularly when they increased in difficulty throughout the task. Further, the harder-over-time (HOT) goals also improved overall sustained attention by not just attenuating the time-on-task vigilance decrement effect, but eliminating it. Those in the goal condition did not slow over time. Interestingly both conditions did have similar TUT experiences and experienced more TUTs over time. This adds further evidence to the above conversation on the divergence of behavioral and subjective measures of attention lapses during sustained attention. Both Robison et al. (2021) and Strayer et al. (2024) posit that attention lapses were reduced due to attentional effort being ramped up after being given specific goals. However, they lack a direct measure of attentional effort, leading to the present study in which we used pupillometry to bridge that gap.

## Attentional effort

The notion of attentional effort being vital for task performance has been investigated for decades and has a marked presence among cognitive-energetic models of task performance (Broadbent, 1971; Hockey et al., 1986; Hockey, 1993, 1997, 2013; Kahneman, 1973; Kanfer & Ackerman, 1989; Sanders, 1983; Shenhav et al., 2017; Unsworth & Robison, 2020; van Zomeren & Brouwer, 1994). Attentional effort refers to the amount of available attentional resources that are being allocated towards a task, thereby determining task engagement. The term also reflects phasic responses in goal-directed arousal (Aston-Jones & Cohen, 2005; Kahneman, 1973). Attentional effort can be influenced by a number of factors, including: motivation, incentives, self-efficacy, personality factors, tonic arousal, task difficulty, and cost/benefit analyses of effort allocation (Langner et al., 2012; Massar et al., 2016; Sadahiani & D’Esposito, 2015; Sturm & Willmes, 2001; Unsworth et al., 2022; van Zomeran & Brouwer, 1994). Essentially, attentional effort regulates how much control is exerted during a given task (Jennings & van der Molen, 2005; Shenhav et al., 2017; Unsworth & Miller, 2021).

Pertinent to the current study, Strayer et al. (2024) provided different goal instructions during a four-choice RT task. A control group received the vague goal to “respond as quickly and accurately as possible.” The goal group received specific timing goals that became harder during each of the three task blocks (i.e., “keep your response times under .450 s while staying as accurate as possible”). The goal group showed a reduction in attention lapses and an overall faster RT distribution. Strayer et al. (2024) suggested the goal effect was partially due to a general effort mobilization and subsequent attentional effort increase compared to the control group. Importantly, this study did not include a direct measure of attentional effort. The current study aimed to replicate and extend Strayer et al. (2024) by incorporating pupillometry to directly assess attentional effort in the context of goal setting.

### Pupillary responses as an index of attentional effort

A considerable amount of research has indicated that pupil dilation fluctuates as a function of the cognitive demands of a task (see Beatty, 1982; Beatty & Lucero-Wagoner, 2000; Kahneman, 1973; Laeng et al., 2012; Mathôt, 2018). This dilation can be explained as a task-evoked pupillary response (TEPR) which indicates a change from baseline pupil size in accordance with increases in attentional effort (intensity; Beatty & Lucero-Wagoner, 2000; Kahneman, 1973; Unsworth & Miller, 2021). These pupillary responses have been associated with activity in the locus-coeruleus norepinephrine (LC-NE) system, which is suggested to play an important role in the regulating of intensity of attention and mobilizing attentional effort as well as sustaining attention and alertness (Aston-Jones & Cohen, 2005; Berridge & Waterhouse, 2003; Gilzenrat et al., 2010; Joshi et al., 2016; Murphy et al., 2014; Rajkowski et al., 1994; Unsworth & Robison, 2017; Varazzani et al., 2015). Recent research has indicated that pupillary responses are also useful for examining attention lapses and changes in sustained attention driven by fluctuations in attentional effort (intensity of attention) corresponding to LC-NE activity (Unsworth & Robison, 2016, 2017; van den Brink et al., 2016). Therefore, we will use pupillary responses to index attentional effort and determine its relationship with the previously observed reduction in attention lapses and boost in overall sustained attention resulting from HOT goal setting (Strayer et al., 2024).

## Current study

The current study used pupillometry methods concurrently with a four-choice RT task to test the hypothesis that progressively harder goals reduce the occurrence of attention lapses and attentional effort is one of the mechanisms behind this effect. This task was used to maintain consistency with prior work (Strayer et al., 2024) and because it has a long-standing history in attention lapse research (e.g., Bertelson & Joffe, 1963; Steinborn et al., 2017; Unsworth et al., 2021).

Consistent with prior research, we assessed lapses in attention behaviorally in terms of examining the slow tail of the RT distribution (e.g., Robison et al., 2021; Strayer et al., 2024; Tse et al., 2010; Unsworth et al., 2010; Unsworth & Robison, 2016). This approach was taken because summary scores can obscure key experimental effects that can be found in different parts of the overall distribution (see Balota & Yap, 2011). As examining the number of lapses/blocks is used in prior research, we also assessed that measure simply for the sake of comparison/completeness (referred to as # (number) of lapses for the rest of the paper; e.g., Bertelson & Joffe, 1963; Bills, 1931, 1935; Broadbent, 1958; Unsworth et al., 2021; Williams et al., 1959). We used thought probes to measure attentional states subjectively. Prior research has used these thought probes to examine how experimental manipulations affect task-related/unrelated thoughts (e.g., Robison et al., 2021; Smallwood & Schooler, 2015; Weinstein, 2018).

We investigated whether setting specific goals that become progressively harder-over-time (HOT) increased attentional effort and decreased attention lapses. In line with goal-setting theory and previous findings (Strayer et al., 2024), we hypothesized that the preparatory and phasic pupil responses in the HOT condition will be more prominent and exhibit less decline over time (as opposed to a control group with a vague goal), indicating that they exerted more attentional effort. Also in line with Strayer et al. (2024), we expected the HOT goal to improve RTs and reduce attention lapses measured behaviorally (via RT distribution & # of lapses) and subjectively (via thought-probes).

## Method

### Participants

Eighty-one students between the ages of 18 and 35 years were recruited from the University of Oregon human subjects pool and were compensated with partial course credit. Subjects were randomly assigned to either the Control condition or the Harder-Over-Time (HOT) goal condition. We aimed to collect a minimum of 40 participants per condition. This sample size provides adequate statistical power to identify medium to large effects (ηp^2^ ≥ .06) both within and between subject. Criteria for exclusion were having any of the following: less than 80% overall task accuracy, less than 50% accuracy in any single block, or a number of attention lapses or average response time over 3 standard deviations above the mean. One participant from the control condition was excluded for excessive # of lapses. This left a final of N = 80 (40 in Control condition, 40 in HOT goal condition; 59 women, 21 men, 0 other/prefer not to say). The task lasted approximately 30 min. Participants provided informed consent and completed a brief demographic survey prior to beginning the task.

### Task

**Fig. 1:**
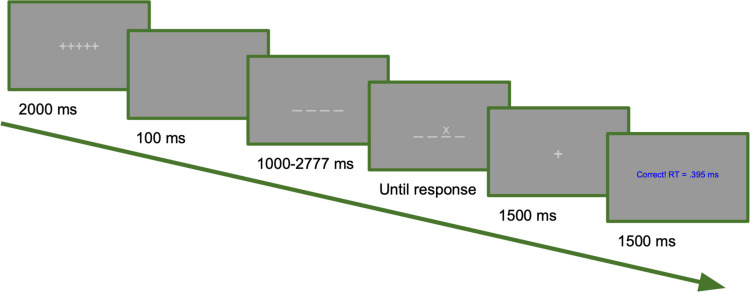
Four-choice response time (RT) task paradigm visual for a single trial

Participants completed a four-choice RT task consisting of 180 trials programmed via E-Prime 2.0 (see Fig. 1). Each trial began on a gray screen with a 2,000 ms light-gray fixation (+++++), followed by a brief blank gray screen for 100 ms, then four light-gray lines appeared in a row in the center of the screen. Following a random time interval (between 1,000 and 2,777 ms), a light-gray target (X) appeared above one of the four lines. Participants responded with a key press corresponding to the location of the target (“F,” “G,” “H,” “J”) and then saw a 1,500 ms fixation screen. Finally, a 1,500 ms feedback screen appeared indicating whether they answered correctly (in blue) or incorrectly (in red). If correct, the RT for that trial and accuracy for the current block was also shown. At five random points in each block, the participant responded to a thought-probe (presented after feedback and prior to beginning the next trial) and then saw a 3,000 ms goal reminder before continuing.

### Pupil measurement

Participants were individually tested in a dark room lit only with ambient screen light. Pupil diameter was continuously binocularly recorded at 120 Hz using a Tobii T300 eye tracker, integrated with a 23-in. monitor (1,920 × 1,080 screen resolution). Subjects were seated approximately 60 cm from the screen with their chin seated in a chin rest. Data from the left eye were used for analyses. Any data points missing due to blinks, off-screen fixations, and/or eye-tracker malfunction were removed. We did not eliminate full trials for missing data points.

### Thought-probes

Participants were randomly presented with 15 thought-probes (five per block) that assessed their attentional state just prior to the probe appearing. The display read:

“Press the key that best describes what you were thinking about just prior to this screen appearing.

1. I was totally focused on the current task
2. I was thinking about my performance on the task
3. I was distracted by sights/sounds in my environment
4. I was thinking about things unrelated to the task
5. My mind was blank.”

Participants responded using the numbers at the top of the keyboard (1–5) corresponding to their answer. Response 1 is coded as on-task, response 2 as task-related, and responses 3–5 as task-unrelated thoughts (TUTs).

### Goal-setting instructions

There were two conditions for this experiment: control (vague goal) and HOT. The control condition first received the instruction: “GOAL: Your goal on this task is to respond as quickly as possible while keeping your accuracy above 95%. Your reaction time and accuracy will be recorded.” Before blocks 2 and 3 they saw: “Remember, your goal on this task is to respond as quickly as possible while keeping your accuracy above 95%.” The same reminder was given after each thought-probe as well. In the HOT condition, the participants were initially instructed: “Your goal on this task is to keep your response times under .450 s while keeping your accuracy above 95%. Your reaction time and accuracy will be recorded.” Before block 2, they saw: “Your goal on this task is to keep your response times under .400 s while keeping your accuracy above 95%. Your reaction time and accuracy will be recorded.” Finally, prior to block 3, they saw: “Your goal on this task is to keep your response times under .350 s while keeping your accuracy above 95%. Your reaction time and accuracy will be recorded.” After each thought-probe, the HOT condition was reminded: “Remember, your goal on this task is to keep your reaction times under [.450, .400, or .350 s] while keeping your accuracy above 95%.”

After completion of the task, participants rated their goal commitment. The question for the control condition was: “How committed were you to the goal of responding as quickly and accurately as possible?” The HOT condition was asked: “How committed were you to the goals of keeping your response time under .450 s, .400 s, and .350 s while staying as accurate as possible?” Subjects provided their response with a keypress between 1 and 7 indicating their goal commitment (anchors: 1 = “Not committed at all”, 4 = “Somewhat committed”, 7 = “Totally committed”).

### Data analyses

#### Behavioral analyses

We set the criteria for excluding individual RTs as RTs outside the range of 150 ms–10 s so anticipatory responses and abnormally long RTs did not skew the results (for consistency with prior work; Strayer et al., 2024). In this experiment, no RTs were excluded based on these criteria (all correct RTs fell within the acceptable range). Only RTs from correct trials included.

The primary measure for behavioral attention lapses was the RT distribution. RTs were ranked from fastest to slowest within-participant and then split into five equal bins (Bin 1 = 20% fastest, Bin 5 = 20% slowest). It is important to consider the entire RT distribution because experimental manipulations can affect different parts of the distribution (i.e., among the slowest RTs; Balota & Yap, 2011). In this case, we expect the largest difference between conditions to occur in Bin 5. The distributions were analyzed with a 2 (condition: Control vs. HOT) × 5 (bin) mixed ANOVA. The # of lapses measure was computed as the number of responses more than 2x the mean RT within-subject. Though we believe it to be a simplistic and less than ideal measure, it was included for the sake of comparison with historical work (e.g., Bertelson & Joffe, 1963; Bills, 1931, 1935; Broadbent, 1958; Unsworth et al., 2021; Williams et al., 1959). We analyzed RT, # of lapses, proportion of TUTs, and accuracy as a function of time using 2 (condition: Control vs. HOT) × 3 (time block) mixed ANOVAs. See Appendix Table 1 for a summary of the *p*-values and effect sizes of the key behavioral analyses.

#### Pupil analyses

The two primary dependent variables were preparatory pupil responses (i.e., prior to target onset) and phasic responses (after target onset). Only trials with a correct response are included in analyses. Preparatory pupil responses were measured as the change from baseline (baseline = the mean pupil diameter from the 2-s fixation period) during the interstimulus interval (ISI) period (i.e., from when the four lines appear until the “X” pops up). The data were split into 70 bins of 40 ms each. Since the ISI varied between 1,000 and 2,777 ms, the later bins contain fewer data. The data were analyzed using a 2 (condition: Control vs. Goal) × 70 (time bin) mixed ANOVA. Phasic pupillary responses were measured as a change from baseline (average of the first 100 ms after the target appears) after the target (“X”) appears. The data were split into 90 bins of 20 ms (1,800 ms total). The data were analyzed using a 2 (condition: Control vs. Goal) × 90 (time bin) mixed ANOVA.

Pupil measures were also examined as a function of time using 2 (condition: Control vs. Goal) × 3 (time block) mixed ANOVAs. See Appendix Table 1 for summary of the *p*-values and effect sizes of the key pupil analyses.

#### Bayesian mixed ANOVA

At the reviewer’s request, we also conducted supplementary Bayesian analyses (see Appendix Table 2 for full details).

## Results

### Behavioral

**Fig. 2:**
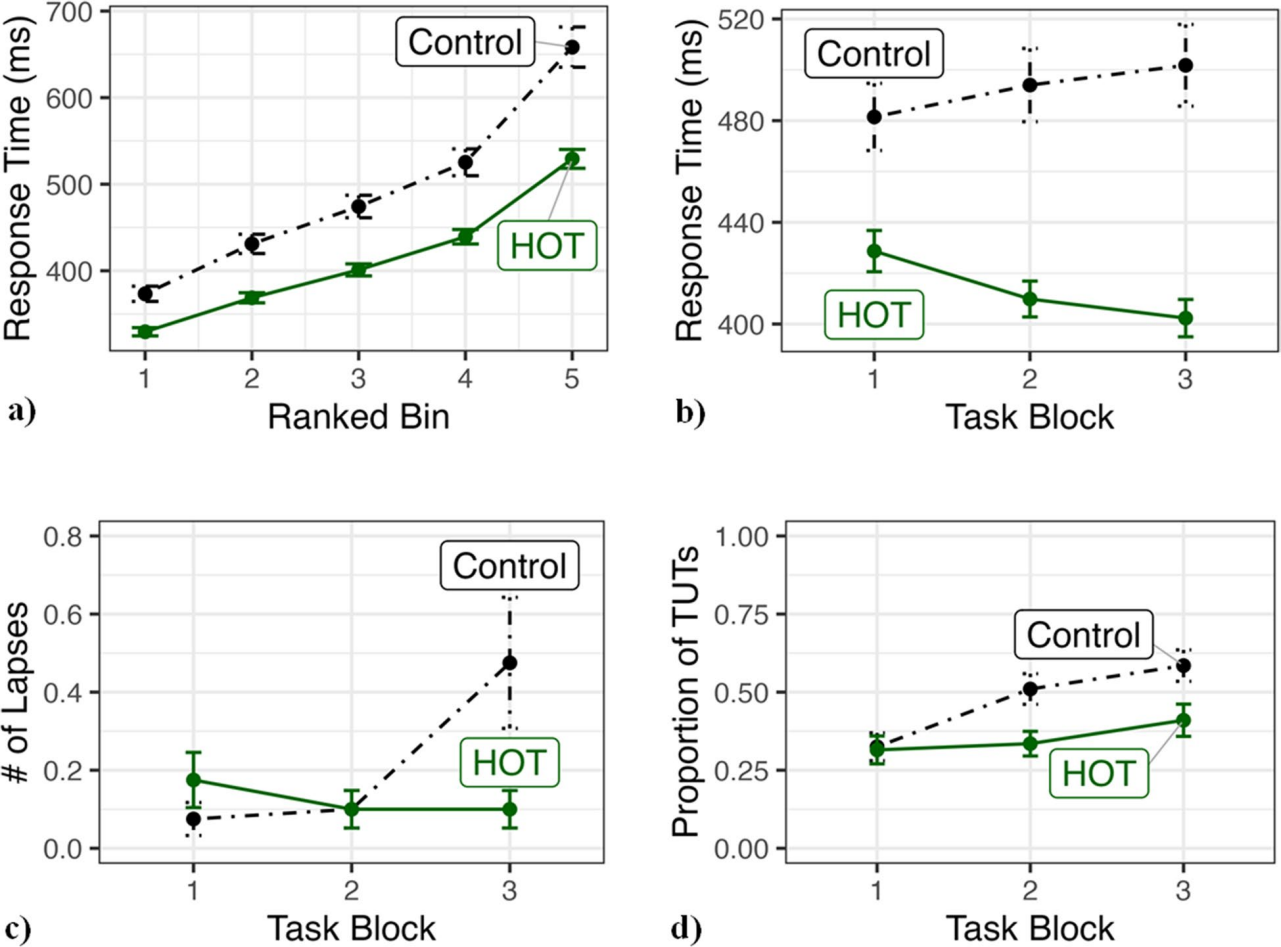
Bars represent 1 standard error. **(a)** Response times (RTs) by condition and ranked bin. Bin = RTs rank-ordered fastest to slowest (1 = fastest, 5 = slowest). Results show those in the control condition had significantly longer slow RTs (bin 5; indicates more attention lapses) and were slower overall. **(b)** RTs by condition and task block. Results show that while the control condition responds slower as time goes on, the harder-over-time (HOT) condition actually responds faster over time. **(c) **Number (#) of lapses by condition and task block. Results show a condition × block interaction wherein the Control condition experiences a higher # of lapses over time compared to the HOT condition which remains relatively stable. **(d)** Proportion of task-unrelated thoughts (TUTs) by condition and task block. Results show a main effect of condition and block, as well as a condition × block interaction. This indicates that the Control group had more TUTs overall, as well as a steeper increase in TUTs over time compared to the HOT condition

The RT distribution, as expected due to the ranking procedure, showed a main effect of bin, *F*(4, 312) = 612.242, *p* < .001, ηp^2^ = .887. Additionally, there was a main effect of condition on response time, *F*(1, 78) = 25.528, *p* < .001, ηp^2^ = .247 (Control *M* = 492.427, *SD* = 87.813 vs. HOT *M* = 413.616, *SD* = 45.061), indicating those in the HOT condition were faster overall. Most importantly, there was a significant bin × condition interaction, *F*(4, 312) = 18.472, *p* < .001, ηp^2^ = .191 (Bin 1: Control *M* = 375.369, *SD* = 55.434 vs. HOT *M* = 329.537, *SD* = 29.978; Bin 5: Control *M* = 658.372, *SD* = 146.9 vs. HOT *M* = 528.263, *SD* = 68.817). This interaction indicates that the largest difference in RTs occurred amongst the slowest RTs (bin 5; see Fig. 2a). This is in line with our hypothesis that subjects given the HOT goal would experience fewer and/or less severe attention lapses throughout the task. To ensure that this effect was not simply due to processing speed, we also performed an ANCOVA to examine differences in Bin 5 between groups while controlling for Bin 1. The HOT goal group still exhibited faster adjusted mean RTs in Bin 5, *F*(1, 77) = 5.021, *p* = .028, *ηp*^*2*^ = .061 (Bin 5: Control *M* = 613.089, *SE* = 11.542 vs. HOT *M* = 574.546, *SE* = 11.542). This indicates that processing speed alone cannot account for the entire effect.

We analyzed RT, # of lapses, proportion of TUTs, and accuracy as a function of time. For RT, there was a significant main effect of condition, *F*(1, 78) = 25.49, *p* < .001, ηp^2^ = .246, and no main effect of block, *F*(2, 156) = .326, *p* = .722. Importantly, there was a condition × block interaction, *F(*2, 156) =14.402, *p* < .001, ηp^2^ = .156. As seen in Fig. 2b, the HOT condition responded quicker over time as the goal became harder, *F*(2, 78) = 22.222, *p* < .001, ηp^2^ = .363. Comparatively, the Control condition showed a significant main effect of block, *F*(2, 78) = 3.384, *p* = .039, ηp^2^ = .08, indicating slower RTs over time (Block 1: Control *M* = 481.469, *SD* = 83.533 vs. HOT *M* = 428.688, *SD* = 51.319; Block 3: Control *M* = 501.76, *SD* = 101.588 vs. HOT *M* = 402.327, *SD* = 46.399).

The ANOVA for # of lapses did not find a main effect of condition, *F*(1, 78) = 1.775, *p* = .187 (Control *M* = .65, *SD* = 1.167; HOT *M* = .38, *SD* = .586), or block, *F*(2, 156) = 2.997, *p* = .053. However, there was a condition × block interaction, *F*(2, 156) = 4.532, *p* = .012, ηp^2^ = .055, showing that while the HOT subjects remained relatively stable in the # of lapses experienced over the course of the task, the Control experienced a significantly higher # of lapses in the final task block (Block 1: Control *M* = .075, *SD* = .267 vs. HOT *M* = .175, *SD* = .447; Block 3: Control *M* = .475, *SD* = 1.062 vs. HOT *M* = .1, *SD* = .304; see Fig. 2c).

The ANOVA for proportion of TUTs over time found a significant main effect for both condition, *F*(1, 78) = 5.2, *p* = .025, ηp^2^ = .063 (Control *M* = .473, *SD* = .248; HOT *M* = .353, *SD* = .222), and block, *F*(2, 156) = 13.141, *p* < .001, ηp^2^ = .144. As well as a condition × block interaction, *F*(2, 156) = 3.755, *p* = .026, ηp^2^ = .046. As seen in Fig. 2d, both groups start with the same proportion of TUTs in the first block, but the Control had a higher proportion of TUTs overall, as well as a steeper increase in TUTs over time (Block 1: Control *M* = .325, *SD* = .282 vs. HOT *M* = .315, *SD* = .282; Block 3: Control *M* = .585, *SD* = .318 vs. HOT *M* = .41, *SD* = .326).

The ANOVA for accuracy found a main effect of condition, *F*(1, 78) = 34.83, *p* < .001, ηp^2^ = .309, wherein the Control group displayed higher accuracy compared to the HOT group (Control *M* = 98.04%, *SD* = .9%; HOT *M* = 95.93%, *SD* = 2.1%). There was no main effect of block, *F*(2, 156) = .786, *p* = .457, or condition × block interaction, *F*(2, 156) = 1.808, *p* = .167. To determine whether the core effects in this study were simply the result of a speed-accuracy trade-off, we decided to re-run the main analyses with overall accuracy as a covariate (see Table 3 in the Appendix). The majority of outcomes were not meaningfully altered; critically, none of the key interaction effects changed, indicating that differences between the two conditions are not just due to a speed-accuracy trade-off.

Goal-commitment scores were significantly higher in the Control condition compared to the HOT group, *F*(1,78) = 10.662, *p* = .002, *ηp*^*2*^ = .12 (Control *M* = 5.9, *SD* = .744; HOT *M* = 5.325 *SD* = .829). See Table 1 in the Appendix for a comparison of the core analyses with and without goal-commitment as a covariable. Most outcomes were not meaningfully changed with the addition. Two analyses for proportion of TUTs were the exception; the results for a main effect of block and the condition × block interaction went from being statistically significant (*p* <.001, *ηp*^*2*^ = .144 and *p* = .026, *ηp*^*2*^ = .046, respectively) to non-significant (*p* = .88, *ηp*^*2*^ = .002 and *p* = .085, *ηp*^*2*^ = .032, respectively) with the goal-commitment covariable. This indicates that a large amount of variance in the TUT measurement can be attributed to the subject’s commitment to achieving the given goals.

### Pupil

**Fig. 3:**
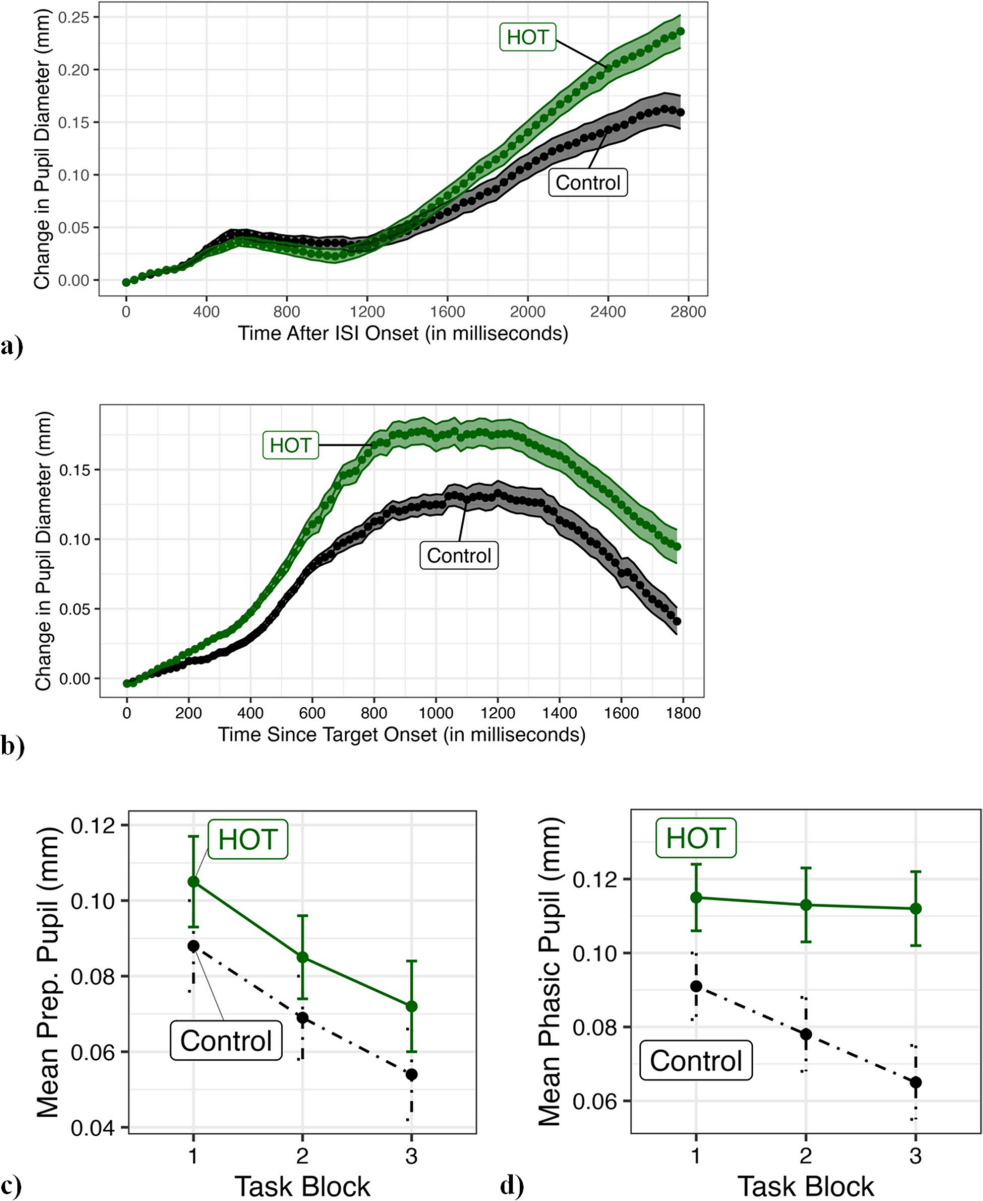
Bars and shaded areas represent 1 standard error. **(a)** Preparatory pupil response (shown as change in pupil diameter over time after interstimulus interval (ISI) onset) by condition. Results showed a main effect of condition and a condition × bin interaction, indicating that the harder-over-time (HOT) condition exerted more attentional effort during the preparatory period. **(b)** Phasic pupil response (shown as change in pupil diameter after target onset). Results found a main effect of condition and a condition × bin interaction. This highlights that the HOT condition had markedly larger phasic responses compared to control. **(c)** Mean preparatory pupil response by condition and task block. Results showed a significant main effect of task block; both groups showed diminished preparatory responses over time. **(d)** Mean phasic response by condition and task block. Although not quite significant, results showed a marginal condition × task block interaction. This suggests tentative evidence that the HOT condition maintained their phasic responses over time while the Control conditions diminished

As expected due to the nature of the preparatory pupil measurement, the results for preparatory pupil did not find a main effect of condition, *F*(1, 78) = 1.372, *p* = .245, ηp^2^ = .017 (Control *M* = .07, *SE* = .01 vs. HOT *M* = .087, *SE* = .01), but did find a main effect of bin, *F*(69, 5382) = 114.232, *p* < .001, ηp^2^ = .594, generally indicating effort being ramped up during the ISI while waiting for the target to appear. More importantly, there was a significant bin × condition interaction, *F*(69, 5382) = 5.07, *p* < .001, ηp^2^ = .061, which indicates those in the HOT condition exerted more attentional effort than those in the control condition (see Fig. 3a). Additionally, there was a main effect of block, *F*(2, 156) = 10.84, *p* < .001, ηp^2^ = .122, but no interaction between block × condition (*p* = .991; Block 1: Control *M* = .088, *SE* = .012 vs. HOT *M* = .105, *SE* = .012; Block 3: Control *M* = .054, *SE* = .012 vs. HOT *M* = .072, *SE* = .012). Both groups showed diminished preparatory responses (arousal) over time, but the HOT group continued to have larger measurements than Control in each block (see Fig. 3c).

Moving to the phasic pupillary responses, we found a significant main effect of condition, *F*(1, 78) = 8.195, *p* = .005, ηp^2^ = .095, and bin × condition interaction, *F*(89, 6942) = 2.021, *p* < .001, ηp^2^ = .025, indicating the HOT condition had markedly larger phasic responses than control (see Fig. 3b; Control *M* = .078, *SE* = .009 vs. HOT *M* = .113, *SE* = .009). Moreover, there was a significant main effect of block, *F*(2, 156) = 4.714, *p* = .01, ηp^2^ = .057, and a not quite significant block × condition interaction, *F*(2, 156) = 2.881, *p* = .059, ηp^2^ = .036 (Block 1: Control *M* = .091, *SE* = .009 vs. HOT *M* = .115, *SE* = .009; Block 3: Control *M* = .065, *SE* = .01 vs. HOT *M* = .112, *SE* = .01). Looking at Fig. 3d, this suggests there is some evidence that while the control condition displayed smaller phasic responses over time, the HOT subjects remained consistent.

### Bayesian mixed ANOVA

Results generally coincide with the core analyses and can be found in Table 2 of the Appendix.

## Results summary

Overall, results demonstrate evidence for our hypothesis that HOT goals partially reduce attention lapses by increasing attentional effort. The pupil analyses indicate participants in the HOT group increased their attentional effort (intensity of attention) more towards the task compared to the Control group. Correspondingly, the HOT group experienced fewer attention lapses in the RT distribution, faster RTs overall (eliminating the vigilance decrement; even responding faster over time), and fewer overall TUTs (with a smaller increase over time vs. control).

## Discussion

We explored the effects of specific goal-setting on attention lapses, attentional effort, and overall sustained attention during an established sustained attention task. Previous evidence indicated that HOT goals reduced the occurrence of attention lapses and mitigated time-on-task effects, possibly due to their influence on attentional effort (Strayer et al., 2024). To test this possibility, we measured pupil fluctuations as a proxy for attentional effort (Beatty, 1982; Beatty & Lucero-Wagoner, 2000; Mathôt, 2018; Unsworth et al., 2020). The current experiment provided participants with either a vague goal or a HOT goal during a four-choice RT task (Bertelson & Joffe, 1963; Steinborn et al., 2017; Strayer et al., 2024; Unsworth et al., 2021). In the pupil results, we found that the goal group exhibited larger preparatory pupil responses compared to control. This indicates they were ramping up their attentional effort (intensity of attention) more when given the HOT goals. Correspondingly, the goal group also exhibited notable larger phasic pupillary responses throughout, and there was some marginal evidence to suggest their phasic response remained relatively consistent over the task even as the control group’s phasic pupil responses diminished (*p* = .059). Taken together, the pupil results indicate that setting HOT goals increased attentional effort toward the task which, in turn, led to higher task engagement and fewer attention lapses. This can be seen in the RT distributions which showed that the HOT group was overall faster. Further, the subjects in the HOT group displayed a reduction in the slow tail of the RT distribution compared to the control condition. This indicates an overall reduction in the amount of particularly slow RTs, suggesting fewer attention lapses were experienced. Looking at the historically used # of lapses measure, the goal group maintained a consistent # of lapses over time while the control group experienced more lapses as the task went on.

This study provides evidence that the HOT goals reduce behaviorally measured attention lapses and increase attentional effort. Meanwhile, the effect on TUTs contributes to a growing amount of research finding differing results when assessing behavioral versus subjective TUT measures (Kucyi et al., 2016; Robison et al., 2021; Steinborn et al., 2017; Strayer et al., 2024; Unsworth et al., 2022). While the measures more or less corresponded in the current study, the agreement of these two measures is far from definitive. For example, Strayer et al. (2024) found that specific goals and HOT goals reduced lapses in the RT distribution but failed to affect the proportion of TUTs experienced during the task. Taken in context with previous research, the current results emphasize the proposition that although behavioral lapses and TUTs may correlate and likely reflect many comparable processes, they are distinct concepts that overlap to different degrees depending on the circumstances (Welhaf & Kane, 2024). Future work must continue to disentangle the commonalities and incongruities between behavioral indicators of lapses and subjective self-reports of task-unrelated thoughts.

The current experiment found that not only did the HOT goals reduce lapses, but they could boost overall sustained attention via time-on-task effects. Looking at RT, the control condition was generally stable over time, just significantly slower overall. In comparison, the HOT condition actually eliminated that typical decrement and became faster over the task as they rose to the challenge of each goal. This is mirrored in the pupil data where we found marginal evidence that phasic responses remained stable over time for the HOT condition instead of diminishing like the control group.

Interestingly, this study found a small difference in accuracy between groups that is consistent with prior findings (Strayer et al., 2024). In previous studies, participants had been given only a speed goal, not an accuracy goal, leading us to believe the difference may be indicative of a speed-accuracy trade-off. In an effort to eliminate the previously found accuracy difference, the current study provided the goal of staying above 95% accuracy to both experimental groups. We found there was a statistically significant difference with the control achieving 98% accuracy versus 95.9% for the HOT goal group (see Appendix Table 3 for summary of main analyses after controlling for accuracy). Even though the HOT goal group had technically lower accuracy, they did collectively achieve the goal of staying above 95%. It is possible that the framing of the speed and accuracy goals caused subjects to differentially interpret the instructions between conditions, encouraging a functional speed-accuracy trade-off for the HOT goal group. That is to say, though the accuracy difference was statistically significant, in many applications there may be no practical distinction between 98% and 96%. If a given task only requires 95% accuracy, then the speed increase may be viewed as more valuable than a marginal accuracy increase. Since the goal group generally met the target for 95% accuracy, they may have been satisfied that that objective was met, and they could therefore focus on speed. Future research should incorporate questions to parse out discrepancies in instruction interpretation between conditions. This could help shed light on whether participants are cognizant of making a tradeoff/decision regarding their speed and accuracy.

Having to engage in repetitive activities is an unavoidable fact of life. These monotonous processes can be vital for safety/efficiency in a number of ways, making it critical to research ways to reduce the occurrence of attention lapses. The utility of this line of research becomes clear when looking at high-risk endeavors like safely directing airplanes, monitoring many children in a pool, or sewing a new kidney into a patient. The research presented here suggests that setting specific goals that become harder over time enhances attentional effort and leads to better sustained attention and fewer attention lapses. Thus, examining goal-setting and other methods that affect attentional effort is a favorable approach when it comes to attention lapse research.

## Conclusion

The current experiments found evidence that setting specific goals that become progressively harder over time increased attentional effort, leading to fewer attention lapses and overall better sustained attention when compared to a vague goal. This includes the elimination of the typical time-on-task effect in RT and mitigates other behavioral and subjective time-on-task effects. Evidence of a TUT effect invites further questions on how to disentangle attention lapses being measured behaviorally versus subjectively. Collectively, the current results suggest that utilizing certain goal-setting techniques can lead individuals to increase attentional effort, resulting in improved task performance and fewer attention lapses.

## Data Availability

Data for this study are available on the Open Science Framework (OSF) at: https://osf.io/m6h8j/. The experiment task file and code are available upon request.

## Appendix A

See Table 1, 2 and 3

**Table 1:** Comparison of p-values and effect sizes before/after using an analysis of covariance to covary out goal commitment for both the original data cleaning procedure and the reviewer-suggested method of removing post-error and post-probe trials

Data-cleaning procedure/analysis	Without goal commitment covariable	With goal commitment covariable
Original data cleaning		
RT × Condition	*p* <.001, *ηp*^*2*^ =.247	*p* <.001, *ηp*^*2*^ =.237
RT Distribution (Bin × Condition interaction)	*p* <.001, *ηp*^*2*^ =.191	*p* <.001, *ηp*^*2*^ =.217*
RT Block (Block × Condition interaction)	*p* <.001, *ηp*^*2*^ =.156	*p* <.001, *ηp*^*2*^ =.175*
# of Lapses × Condition	*p* =.187, *ηp*^*2*^ =.022	*p* =.072, *ηp*^*2*^ =.041
# of Lapses (Block × Condition interaction)	*p* =.012, *ηp*^*2*^ =.055	*p* =.029, *ηp*^*2*^ =.045
Prop. of TUTs × Condition	*p* =.025, *ηp*^*2*^ =.063	*p* =.009, *ηp*^*2*^ =.085*
Prop. of TUTs × Block	*p* <.001, *ηp*^*2*^ =.144	*p* =.88, *ηp*^*2*^ =.002^**+**^
Prop. of TUTs (Block × Condition interaction)	*p* =.026, *ηp*^*2*^ =.046	*p* =.085, *ηp*^*2*^ =.032^**+**^
Prep. Pupil × Condition	*p* =.245, *ηp*^*2*^ =.017	*p* =.291, *ηp*^*2*^ =.014
Prep. Pupil × Block	*p* <.001, *ηp*^*2*^ =.122	*p* =.234, *ηp*^*2*^ =.019^**+**^
Prep. Pupil (Bin × Condition interaction)	*p* <.001, *ηp*^*2*^ =.061	*p* <.001, *ηp*^*2*^ =.066*
Phasic Pupil × Condition	*p =*.005, *ηp*^*2*^ =.095	*p =*.012, *ηp*^*2*^ =.079
Phasic Pupil (Bin × Condition interaction)	*p* <.001, *ηp*^*2*^ =.025	*p* <.001, *ηp*^*2*^ =.02
Phasic Pupil (Block × Condition interaction)	*p =*.059, *ηp*^*2*^ =.036	*p =*.11, *ηp*^*2*^ =.028
Accuracy × Condition	*p* <.001, *ηp*^*2*^ =.309	*p* <.001, *ηp*^*2*^ =.246
Accuracy (Block × Condition interaction)	*p =*.167, *ηp*^*2*^ =.023	*p =*.142, *ηp*^*2*^ =.025
Post-error + post-probe trials removed		
RT × Condition	*p* <.001, *ηp*^*2*^ =.249^**^^**^	*p* <.001, *ηp*^*2*^ =.240
RT Distribution (Bin × Condition interaction)	*p* <.001, *ηp*^*2*^ =.199^**^^**^	*p* <.001, *ηp*^*2*^ =.225
RT Block (Block × Condition interaction)	*p* <.001, *ηp*^*2*^ =.198^**^^**^	*p* <.001, *ηp*^*2*^ =.166
# of Lapses × Condition	*p* =.147, *ηp*^*2*^ =.027	*p* =.057, *ηp*^*2*^ =.046
# of Lapses (Block × Condition interaction)	*p* =.014, *ηp*^*2*^ =.053	*p* =.024, *ηp*^*2*^ =.047
Prep. Pupil × Condition	*p* =.22, *ηp*^*2*^ =.019	*p* =.303, *ηp*^*2*^ =.014
Prep. Pupil × Block	*p* <.001, *ηp*^*2*^ =.12	*p* =.169, *ηp*^*2*^ =.023^**+**^
Prep. Pupil (Bin × Condition interaction)	*p* <.001, *ηp*^*2*^ =.074^**^^**^	*p* <.001, *ηp*^*2*^ =.075
Phasic Pupil × Condition	*p =*.007, *ηp*^*2*^ =.091	*p =*.014, *ηp*^*2*^ =.076
Phasic Pupil (Bin × Condition interaction)	*p* <.001, *ηp*^*2*^ =.025	*p* <.001, *ηp*^*2*^ =.021^**^^**^
Phasic Pupil (Block × Condition interaction)	*p =*.081, *ηp*^*2*^ =.032	*p =*.108, *ηp*^*2*^ =.028

***** = Analyses where including the goal commitment covariable either increased an already statistically significant effect size, or the result became statistically significant when it was previously not significant. ^**+**^ = Analyses that became statistically insignificant when including the goal commitment covariable when they were previously significant. ^**^^**^ = Analyses where removing post-error and post-probe trials increased an already statistically significant effect size. No significant results became insignificant due to the removal of post-error and post-probe trials

*RT* response time, *Prop.* proportion, *TUTs* task-unrelated thoughts, *Prep.* Preparatory

**Table 2:** Results for Bayes factors analyses with default r =.5 and the range of values across priors r = {.3, .5, .707, 1}, as a robustness check

DV	Effect	BF_10_	BF_10_ range
RT distribution	Condition	4,355.482	2,808–6,327
	Bin	>100,000	>100,000
	Condition × Bin	>100,000	>100,000
RT (block)	Condition	3,711.790	2,236–6,201
	Block	.057	.016–.119
	Condition × Block	9,175.682	6,595–9,176
# of Lapses	Condition	.310	.166–.477
	Block	.782	.272–1.334
	Condition × Block	5.437	2.624–6.139
TUTs	Condition	2.148	1.494–2.402
	Block	2,203.557	1,297–2,281
	Condition × Block	1.875	.759–2.362
Prep. Pupil	Condition	.369	.206–.535
	Block	>100,000	>100,000
	Condition × Block	.002	0–.005
	Condition × Bin	>100,000	>100,000
Phasic Pupil	Condition	.379	.197–.551
	Block	>100,000	>100,000
	Condition × Block	.002	.000–.005
	Condition × Bin	>100,000	>100,000

At the request of a reviewer, we assessed evidence for effects and interactions of interest using Bayes factors (BF₁₀). They were derived from nested model comparisons, with BF₁₀ indicating the likelihood of the data under a model including the named effect (plus lower-order terms) against an otherwise identical model excluding that effect. BF₁₀s were computed with the *BayesFactor* package in R using JZS priors (default; fixed-effect scale rscaleFixed = 0.50; Morey & Rouder, 2018). Robustness checks varied the prior width (r = 0.30, 0.50, 0.707, 1.00). We report the range of BF₁₀ (evidence for models including the effect vs. excluding it) across all priors; values > 3 indicate at least moderate evidence for inclusion, values <.33 indicate moderate evidence for exclusion. For the RT distribution, we fit a mixed ANOVA with condition (between subjects) and bin (within subjects), treating subject as a random effect (random intercept). For the RT, # of lapses, and proportion of TUTs, we fit a mixed ANOVA with condition (between subjects) and time block (within subjects), treating subject as a random effect (random intercept). For preparatory and phasic pupil responses, we fit a mixed ANOVA with condition (between subjects) and time block × bin (within subjects), treating subject as a random effect (random intercept)

*RT* response time, *Prop.* proportion, *TUTs* task-unrelated thoughts, *Prep.* Preparatory

**Table 3:** A Comparison of p-values and effect sizes before/after using an analysis of covariance to covary out overall accuracy

Analysis	Without accuracy covariable	With accuracy covariable
RT (Main effect of Condition)	*p* <.001, *ηp*^*2*^ =.247	*p* <.001, *ηp*^*2*^ =.154
RT Distribution (Bin × Condition interaction)	*p* <.001, *ηp*^*2*^ =.191	*p* <.001, *ηp*^*2*^ =.152
RT Block (Block × Condition interaction)	*p* <.001, *ηp*^*2*^ =.156	*p* <.001, *ηp*^*2*^ =.140
# of Lapses (Main effect of Condition)	*p* =.187, *ηp*^*2*^ =.022	*p* =.091, *ηp*^*2*^ =.037
# of Lapses (Block × Condition interaction)	*p* =.012, *ηp*^*2*^ =.055	*p* =.015, *ηp*^*2*^ =.053
Proportion of TUTs (Main effect of Condition)	*p* =.025, *ηp*^*2*^ =.063	*p* =.007, *ηp*^*2*^ =.09
Proportion of TUTs (Main effect of Block)	*p* <.001, *ηp*^*2*^ =.144	*p* =.86, *ηp*^*2*^ =.002^**+**^
Prop. of TUTs (Block × Condition interaction)	*p* =.026, *ηp*^*2*^ =.046	*p* =.044, *ηp*^*2*^ =.040
Preparatory Pupil (Main effect of Condition)	*p* =.245, *ηp*^*2*^ =.017	*p* =.571, *ηp*^*2*^ =.004
Preparatory Pupil (Main effect of Block)	*p* <.001, *ηp*^*2*^ =.122	*p* =.409, *ηp*^*2*^ =.012^**+**^
Preparatory Pupil (Bin × Condition interaction)	*p* <.001, *ηp*^*2*^ =.061	*p* <.001, *ηp*^*2*^ =.056
Phasic Pupil (Main effect of Condition)	*p =*.005, *ηp*^*2*^ =.095	*p =*.004, *ηp*^*2*^ =.102
Phasic Pupil (Bin × Condition interaction)	*p* <.001, *ηp*^*2*^ =.025	*p* <.001, *ηp*^*2*^ =.037
Phasic Pupil (Block × Condition interaction)	*p =*.059, *ηp*^*2*^ =.036	*p =*.277, *ηp*^*2*^ =.017

^**+**^ = Analyses that became statistically insignificant when including the accuracy covariable when they were previously significant

*RT* response time, *Prop.* proportion, *TUTs* task-unrelated thoughts, *Prep.* preparatory

## Code availability

Analyses were performed using SPSS GUI, but the code can be generated upon request.

## Declarations

### Open practices statement

This study was not preregistered and the data will be made available on the Open Science Framework at: https://osf.io/m6h8j/. Other materials are available upon request.
